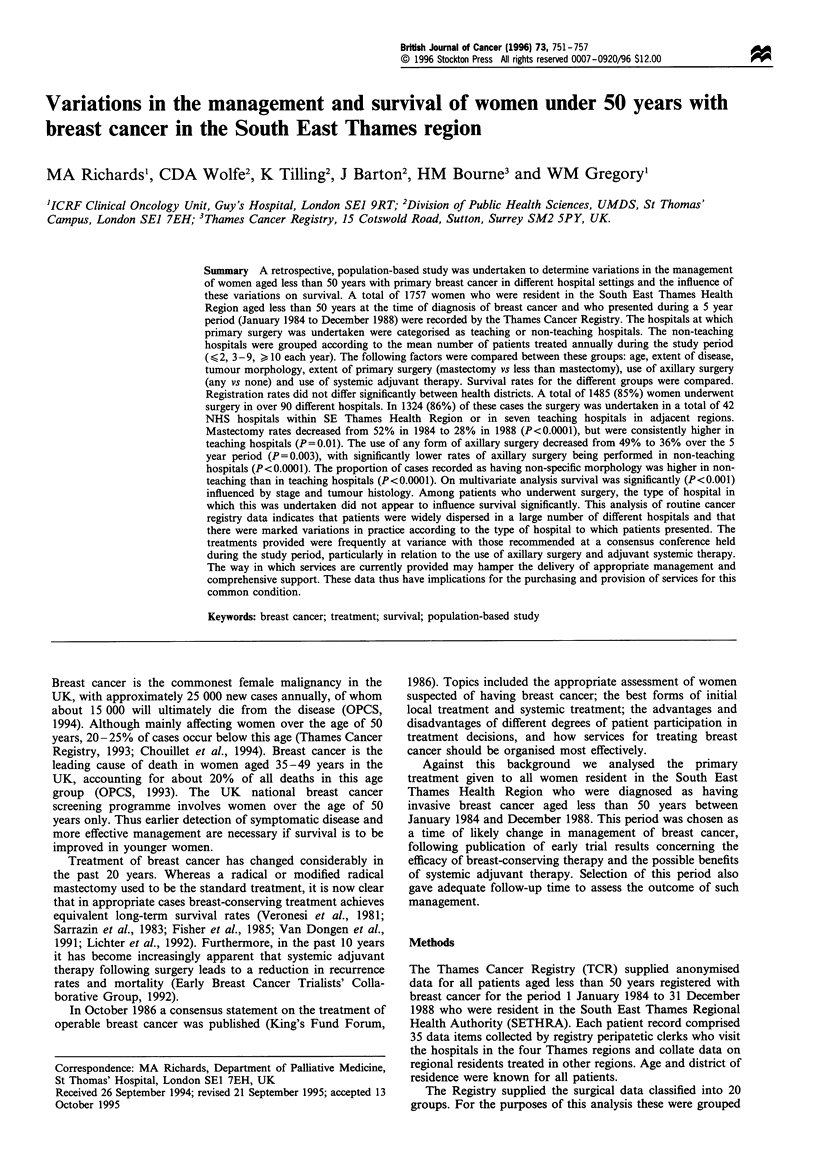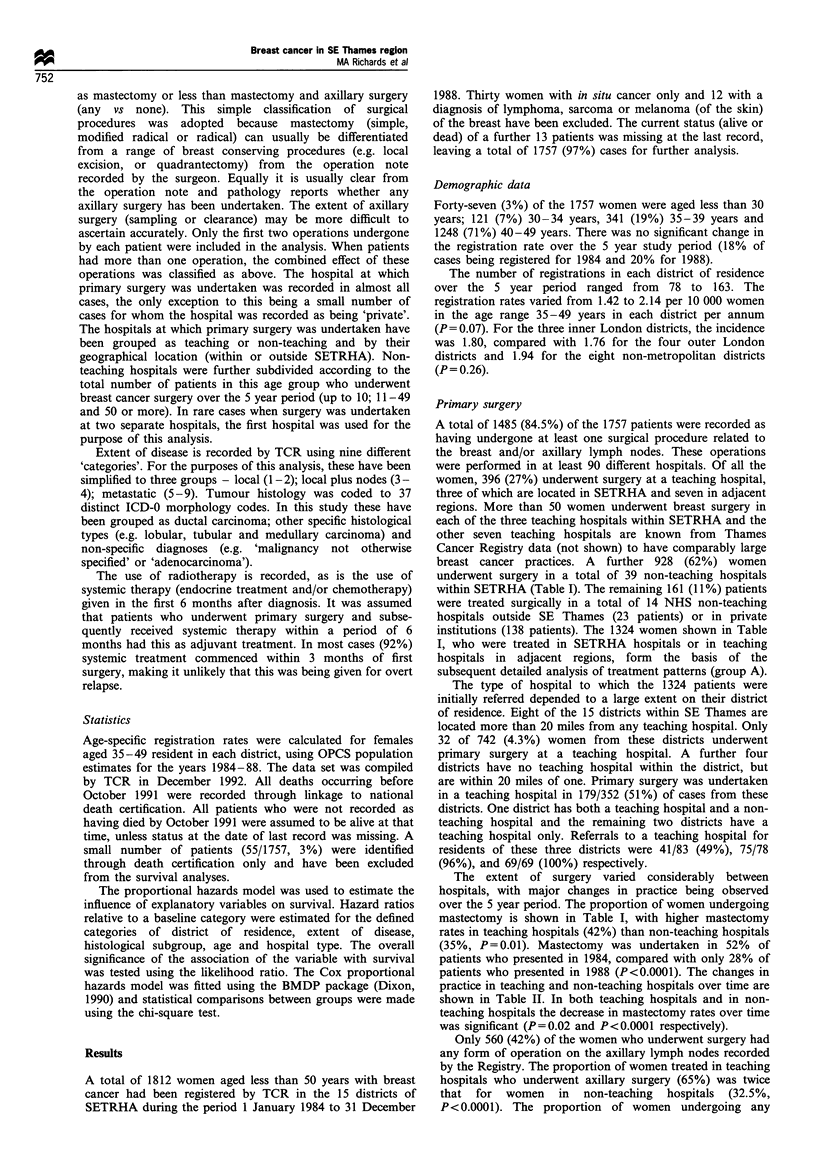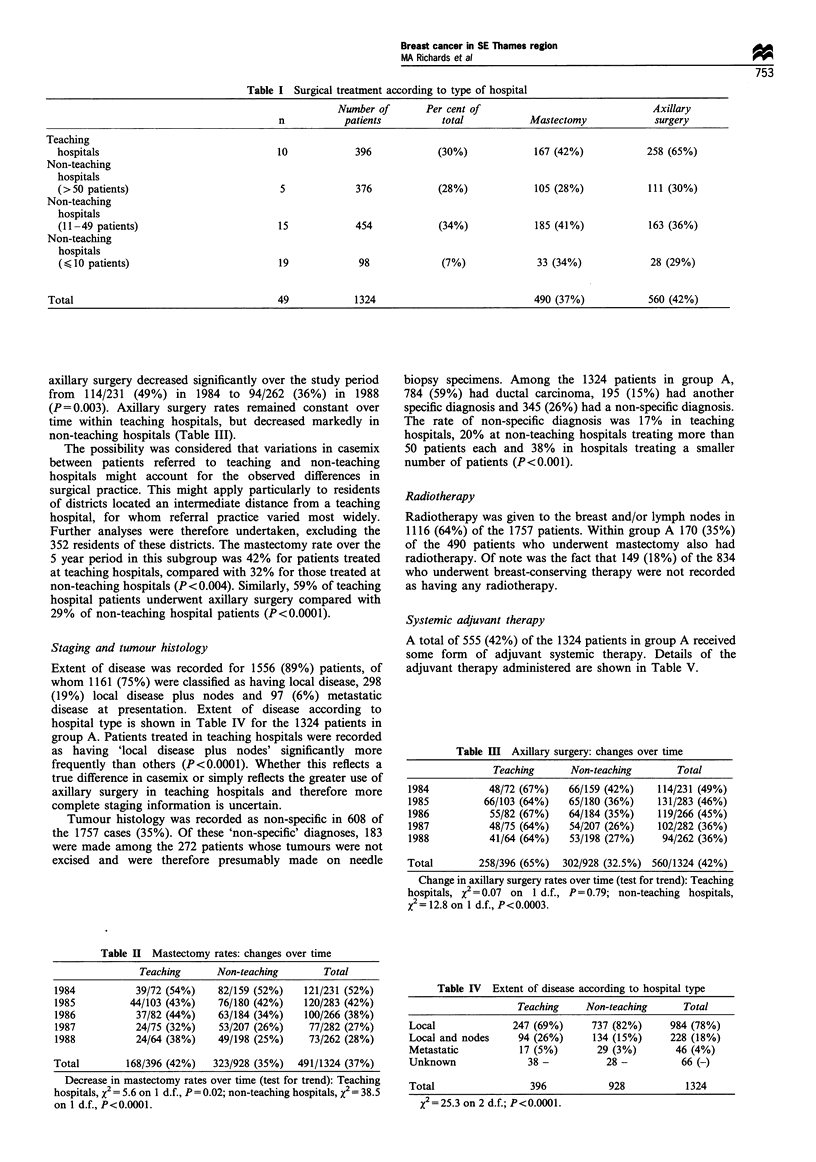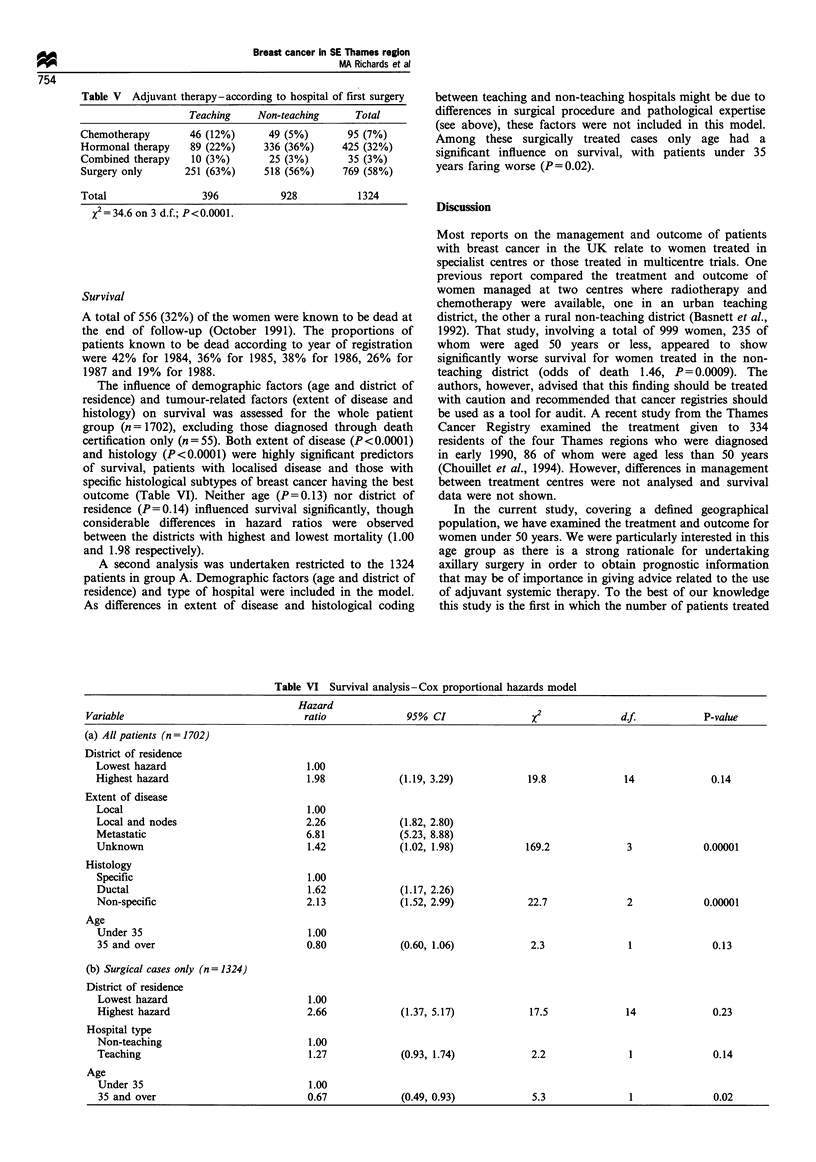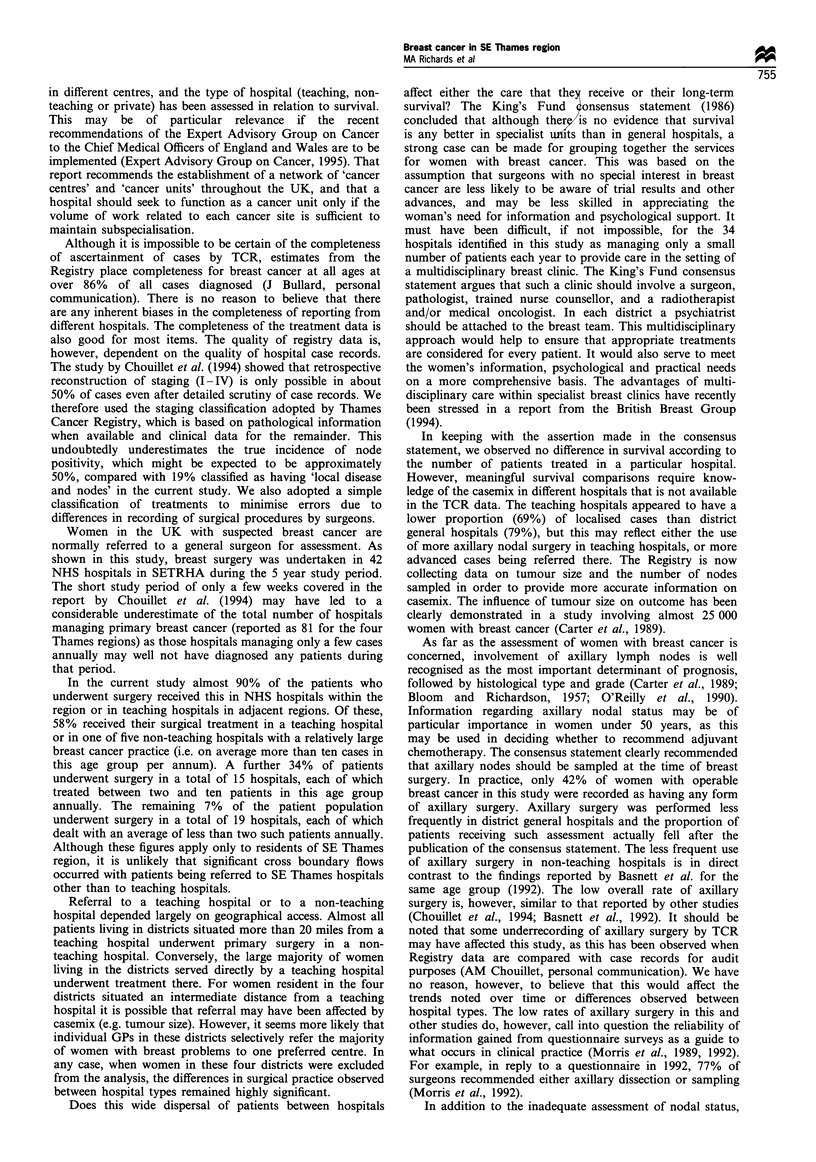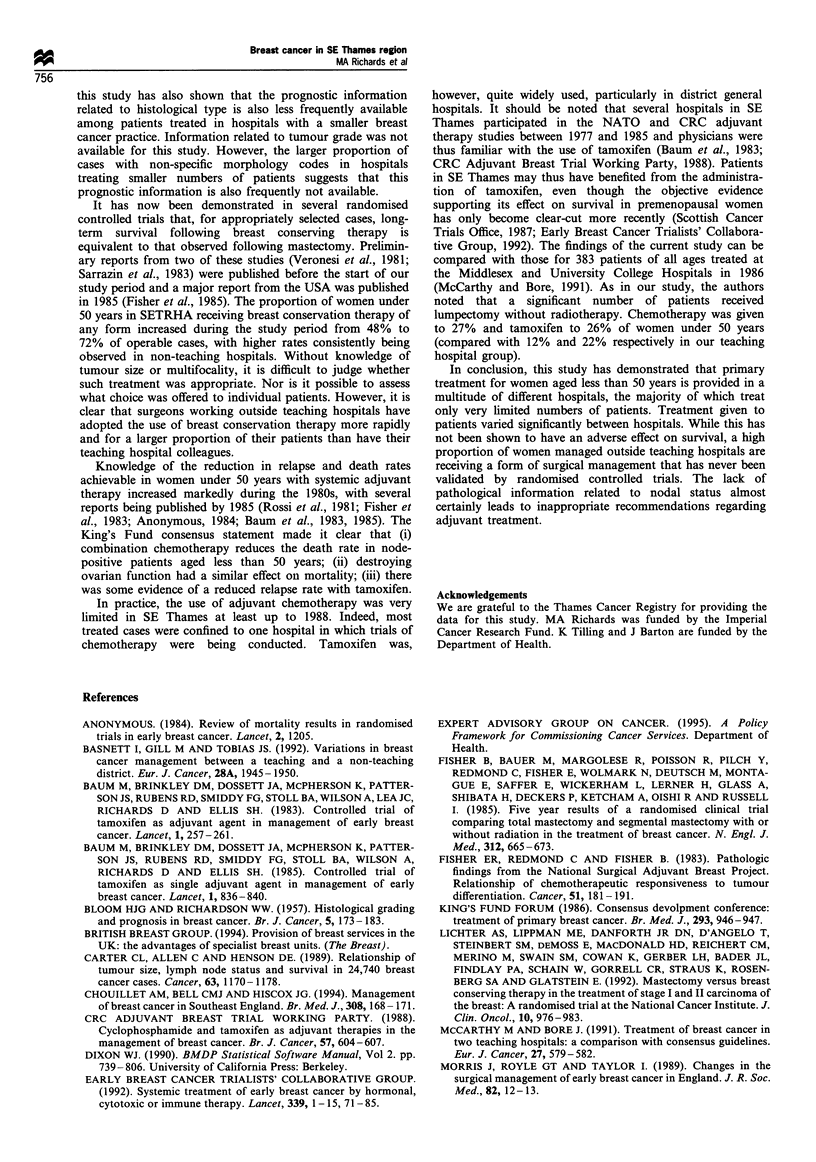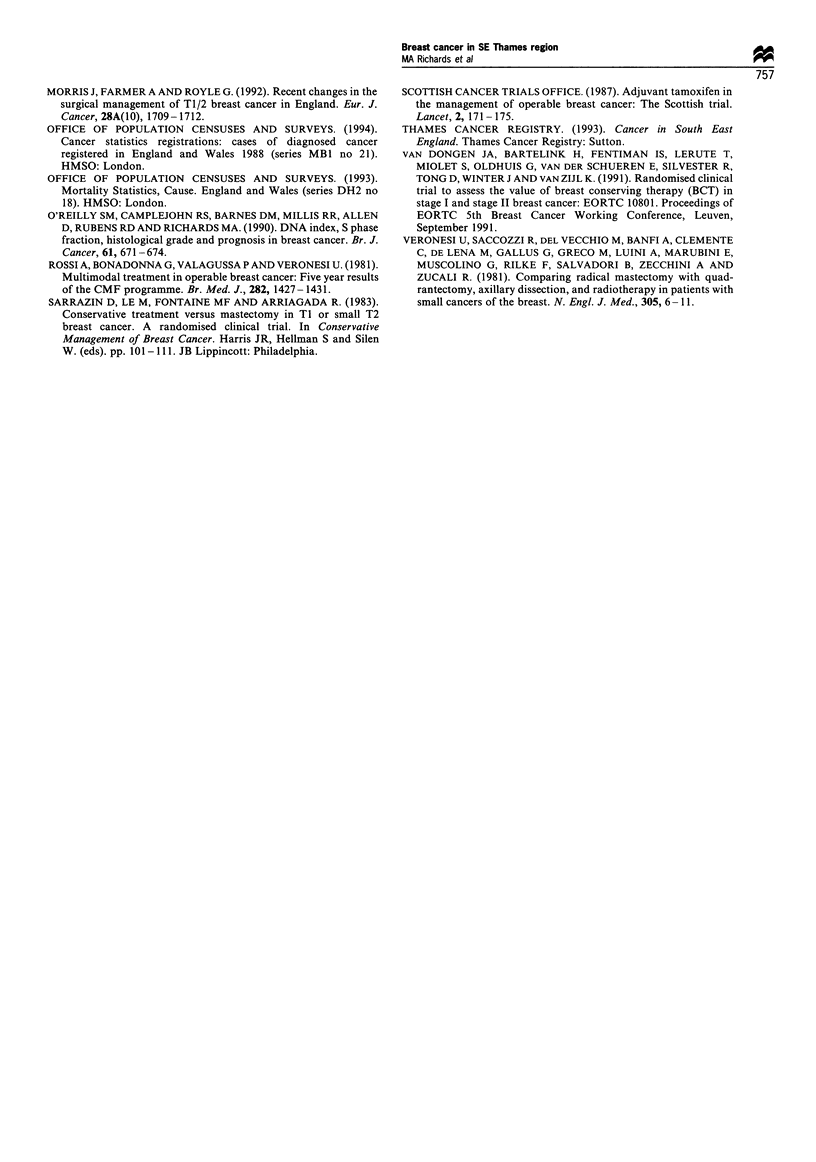# Variations in the management and survival of women under 50 years with breast cancer in the South East Thames region.

**DOI:** 10.1038/bjc.1996.131

**Published:** 1996-03

**Authors:** M. A. Richards, C. D. Wolfe, K. Tilling, J. Barton, H. M. Bourne, W. M. Gregory

**Affiliations:** ICRF Clinical Oncology Unit, Guy's Hospital, London, UK.

## Abstract

A retrospective, population-based study was undertaken to determine variations in the management of women aged less than 50 years with primary breast cancer in different hospital settings and the influence of these variations on survival. A total of 1757 women who were resident in the South East Thames Health Region aged less than 50 years at the time of diagnosis of breast cancer and who presented during a 5 year period (January 1984 to December 1988) were recorded by the Thames Cancer Registry. The hospitals at which primary surgery was undertaken were categorised as teaching or non-teaching hospitals. The non-teaching hospitals were grouped according to the mean number of patients treated annually during the study period (< or = 2, 3-9, > or = 10 each year). The following factors were compared between these groups: age, extent of disease, tumour morphology, extent of primary surgery (mastectomy vs less than mastectomy), use of axillary surgery (any vs none) and use of systemic adjuvant therapy. Survival rates for the different groups were compared. Registration rates did not differ significantly between health districts. A total of 1485 (85%) women underwent surgery in over 90 different hospitals. In 1324 (86%) of these cases the surgery was undertaken in a total of 42 NHS hospitals within SE Thames Health Region or in seven teaching hospitals in adjacent regions. Mastectomy rates decreased from 52% in 1984 to 28% in 1988 (P<0.0001), but were consistently higher in teaching hospitals (P=0.01). The use of any form of axillary surgery decreased from 49% to 36% over the 5 year period (P=0.003), with significantly lower rates of axillary surgery being performed in non-teaching hospitals (P<0.0001). The proportion of cases recorded as having non-specific morphology was higher in nonteaching than in teaching hospitals (P<0.0001). On multivariate analysis survival was significantly (P<0.001) influenced by stage and tumour histology. Among patients who underwent surgery, the type of hospital in which this was undertaken did not appear to influence survival significantly. This analysis of routine cancer registry data indicates that patients were widely dispersed in a large number of different hospitals and that there were marked variations in practice according to the type of hospital to which patients presented. The treatments provided were frequently at variance with those recommended at a consensus conference held during the study period, particularly in relation to the use of axillary surgery and adjuvant systemic therapy. The way in which services are currently provided may hamper the delivery of appropriate management and comprehensive support. These data thus have implications for the purchasing and provision of services for this common condition.


British Journal of Cancer (1996) 73, 751-757
© 1996 Stockton Press All rights reserved 0007-0920/96 $12.00

### Variations in the management and survival of women under 50 years with breast cancer in the South East Thames region

MA Richards[1], CDA Wolfe[2], K Tilling[2], J Barton[2], HM Bourne[3] and WM Gregory[1]

*[1]ICRF Clinical Oncology Unit, Guy's Hospital, London SE1 9RT; [2]Division of Public Health Sciences, UMDS, St Thomas' Campus, London SE1 7EH; [3]Thames Cancer Registry, 15 Cotswold Road, Sutton, Surrey SM2 5PY, UK.*

**Summary** A retrospective, population-based study was undertaken to determine variations in the management of women aged less than 50 years with primary breast cancer in different hospital settings and the influence of these variations on survival. A total of 1757 women who were resident in the South East Thames Health Region aged less than 50 years at the time of diagnosis of breast cancer and who presented during a 5 year period (January 1984 to December 1988) were recorded by the Thames Cancer Registry. The hospitals at which primary surgery was undertaken were categorised as teaching or non-teaching hospitals. The non-teaching hospitals were grouped according to the mean number of patients treated annually during the study period ($\leqslant 2$, 3–9, $\geqslant 10$ each year). The following factors were compared between these groups: age, extent of disease, tumour morphology, extent of primary surgery (mastectomy *vs* less than mastectomy), use of axillary surgery (any *vs* none) and use of systemic adjuvant therapy. Survival rates for the different groups were compared. Registration rates did not differ significantly between health districts. A total of 1485 (85%) women underwent surgery in over 90 different hospitals. In 1324 (86%) of these cases the surgery was undertaken in a total of 42 NHS hospitals within SE Thames Health Region or in seven teaching hospitals in adjacent regions. Mastectomy rates decreased from 52% in 1984 to 28% in 1988 ($P<0.0001$), but were consistently higher in teaching hospitals ($P=0.01$). The use of any form of axillary surgery decreased from 49% to 36% over the 5 year period ($P=0.003$), with significantly lower rates of axillary surgery being performed in non-teaching hospitals ($P<0.0001$). The proportion of cases recorded as having non-specific morphology was higher in non-teaching than in teaching hospitals ($P<0.0001$). On multivariate analysis survival was significantly ($P<0.001$) influenced by stage and tumour histology. Among patients who underwent surgery, the type of hospital in which this was undertaken did not appear to influence survival significantly. This analysis of routine cancer registry data indicates that patients were widely dispersed in a large number of different hospitals and that there were marked variations in practice according to the type of hospital to which patients presented. The treatments provided were frequently at variance with those recommended at a consensus conference held during the study period, particularly in relation to the use of axillary surgery and adjuvant systemic therapy. The way in which services are currently provided may hamper the delivery of appropriate management and comprehensive support. These data thus have implications for the purchasing and provision of services for this common condition.

**Keywords:** breast cancer; treatment; survival; population-based study

Breast cancer is the commonest female malignancy in the UK, with approximately 25 000 new cases annually, of whom about 15 000 will ultimately die from the disease (OPCS, 1994). Although mainly affecting women over the age of 50 years, 20–25% of cases occur below this age (Thames Cancer Registry, 1993; Chouillet *et al.*, 1994). Breast cancer is the leading cause of death in women aged 35–49 years in the UK, accounting for about 20% of all deaths in this age group (OPCS, 1993). The UK national breast cancer screening programme involves women over the age of 50 years only. Thus earlier detection of symptomatic disease and more effective management are necessary if survival is to be improved in younger women.

Treatment of breast cancer has changed considerably in the past 20 years. Whereas a radical or modified radical mastectomy used to be the standard treatment, it is now clear that in appropriate cases breast-conserving treatment achieves equivalent long-term survival rates (Veronesi *et al.*, 1981; Sarrazin *et al.*, 1983; Fisher *et al.*, 1985; Van Dongen *et al.*, 1991; Lichter *et al.*, 1992). Furthermore, in the past 10 years it has become increasingly apparent that systemic adjuvant therapy following surgery leads to a reduction in recurrence rates and mortality (Early Breast Cancer Trialists' Collaborative Group, 1992).

In October 1986 a consensus statement on the treatment of operable breast cancer was published (King's Fund Forum, 1986). Topics included the appropriate assessment of women suspected of having breast cancer; the best forms of initial local treatment and systemic treatment; the advantages and disadvantages of different degrees of patient participation in treatment decisions, and how services for treating breast cancer should be organised most effectively.

Against this background we analysed the primary treatment given to all women resident in the South East Thames Health Region who were diagnosed as having invasive breast cancer aged less than 50 years between January 1984 and December 1988. This period was chosen as a time of likely change in management of breast cancer, following publication of early trial results concerning the efficacy of breast-conserving therapy and the possible benefits of systemic adjuvant therapy. Selection of this period also gave adequate follow-up time to assess the outcome of such management.

### Methods

The Thames Cancer Registry (TCR) supplied anonymised data for all patients aged less than 50 years registered with breast cancer for the period 1 January 1984 to 31 December 1988 who were resident in the South East Thames Regional Health Authority (SETHRA). Each patient record comprised 35 data items collected by registry peripatetic clerks who visit the hospitals in the four Thames regions and collate data on regional residents treated in other regions. Age and district of residence were known for all patients.

The Registry supplied the surgical data classified into 20 groups. For the purposes of this analysis these were grouped

Correspondence: MA Richards, Department of Palliative Medicine, St Thomas' Hospital, London SE1 7EH, UK
Received 26 September 1994; revised 21 September 1995; accepted 13 October 1995

as mastectomy or less than mastectomy and axillary surgery (any *vs* none). This simple classification of surgical procedures was adopted because mastectomy (simple, modified radical or radical) can usually be differentiated from a range of breast conserving procedures (e.g. local excision, or quadrantectomy) from the operation note recorded by the surgeon. Equally it is usually clear from the operation note and pathology reports whether any axillary surgery has been undertaken. The extent of axillary surgery (sampling or clearance) may be more difficult to ascertain accurately. Only the first two operations undergone by each patient were included in the analysis. When patients had more than one operation, the combined effect of these operations was classified as above. The hospital at which primary surgery was undertaken was recorded in almost all cases, the only exception to this being a small number of cases for whom the hospital was recorded as being 'private'. The hospitals at which primary surgery was undertaken have been grouped as teaching or non-teaching and by their geographical location (within or outside SETRHA). Non-teaching hospitals were further subdivided according to the total number of patients in this age group who underwent breast cancer surgery over the 5 year period (up to 10; 11–49 and 50 or more). In rare cases when surgery was undertaken at two separate hospitals, the first hospital was used for the purpose of this analysis.

Extent of disease is recorded by TCR using nine different 'categories'. For the purposes of this analysis, these have been simplified to three groups – local (1–2); local plus nodes (3–4); metastatic (5–9). Tumour histology was coded to 37 distinct ICD-0 morphology codes. In this study these have been grouped as ductal carcinoma; other specific histological types (e.g. lobular, tubular and medullary carcinoma) and non-specific diagnoses (e.g. 'malignancy not otherwise specified' or 'adenocarcinoma').

The use of radiotherapy is recorded, as is the use of systemic therapy (endocrine treatment and/or chemotherapy) given in the first 6 months after diagnosis. It was assumed that patients who underwent primary surgery and subsequently received systemic therapy within a period of 6 months had this as adjuvant treatment. In most cases (92%) systemic treatment commenced within 3 months of first surgery, making it unlikely that this was being given for overt relapse.

### *Statistics*

Age-specific registration rates were calculated for females aged 35–49 resident in each district, using OPCS population estimates for the years 1984–88. The data set was compiled by TCR in December 1992. All deaths occurring before October 1991 were recorded through linkage to national death certification. All patients who were not recorded as having died by October 1991 were assumed to be alive at that time, unless status at the date of last record was missing. A small number of patients (55/1757, 3%) were identified through death certification only and have been excluded from the survival analyses.

The proportional hazards model was used to estimate the influence of explanatory variables on survival. Hazard ratios relative to a baseline category were estimated for the defined categories of district of residence, extent of disease, histological subgroup, age and hospital type. The overall significance of the association of the variable with survival was tested using the likelihood ratio. The Cox proportional hazards model was fitted using the BMDP package (Dixon, 1990) and statistical comparisons between groups were made using the chi-square test.

### Results

A total of 1812 women aged less than 50 years with breast cancer had been registered by TCR in the 15 districts of SETRHA during the period 1 January 1984 to 31 December 1988. Thirty women with *in situ* cancer only and 12 with a diagnosis of lymphoma, sarcoma or melanoma (of the skin) of the breast have been excluded. The current status (alive or dead) of a further 13 patients was missing at the last record, leaving a total of 1757 (97%) cases for further analysis.

### *Demographic data*

Forty-seven (3%) of the 1757 women were aged less than 30 years; 121 (7%) 30–34 years, 341 (19%) 35–39 years and 1248 (71%) 40–49 years. There was no significant change in the registration rate over the 5 year study period (18% of cases being registered for 1984 and 20% for 1988).

The number of registrations in each district of residence over the 5 year period ranged from 78 to 163. The registration rates varied from 1.42 to 2.14 per 10 000 women in the age range 35–49 years in each district per annum ($P = 0.07$). For the three inner London districts, the incidence was 1.80, compared with 1.76 for the four outer London districts and 1.94 for the eight non-metropolitan districts ($P = 0.26$).

### *Primary surgery*

A total of 1485 (84.5%) of the 1757 patients were recorded as having undergone at least one surgical procedure related to the breast and/or axillary lymph nodes. These operations were performed in at least 90 different hospitals. Of all the women, 396 (27%) underwent surgery at a teaching hospital, three of which are located in SETRHA and seven in adjacent regions. More than 50 women underwent breast surgery in each of the three teaching hospitals within SETRHA and the other seven teaching hospitals are known from Thames Cancer Registry data (not shown) to have comparably large breast cancer practices. A further 928 (62%) women underwent surgery in a total of 39 non-teaching hospitals within SETRHA (Table I). The remaining 161 (11%) patients were treated surgically in a total of 14 NHS non-teaching hospitals outside SE Thames (23 patients) or in private institutions (138 patients). The 1324 women shown in Table I, who were treated in SETRHA hospitals or in teaching hospitals in adjacent regions, form the basis of the subsequent detailed analysis of treatment patterns (group A).

The type of hospital to which the 1324 patients were initially referred depended to a large extent on their district of residence. Eight of the 15 districts within SE Thames are located more than 20 miles from any teaching hospital. Only 32 of 742 (4.3%) women from these districts underwent primary surgery at a teaching hospital. A further four districts have no teaching hospital within the district, but are within 20 miles of one. Primary surgery was undertaken in a teaching hospital in 179/352 (51%) of cases from these districts. One district has both a teaching hospital and a non-teaching hospital and the remaining two districts have a teaching hospital only. Referrals to a teaching hospital for residents of these three districts were 41/83 (49%), 75/78 (96%), and 69/69 (100%) respectively.

The extent of surgery varied considerably between hospitals, with major changes in practice being observed over the 5 year period. The proportion of women undergoing mastectomy is shown in Table I, with higher mastectomy rates in teaching hospitals (42%) than non-teaching hospitals (35%, $P = 0.01$). Mastectomy was undertaken in 52% of patients who presented in 1984, compared with only 28% of patients who presented in 1988 ($P < 0.0001$). The changes in practice in teaching and non-teaching hospitals over time are shown in Table II. In both teaching hospitals and in non-teaching hospitals the decrease in mastectomy rates over time was significant ($P = 0.02$ and $P < 0.0001$ respectively).

Only 560 (42%) of the women who underwent surgery had any form of operation on the axillary lymph nodes recorded by the Registry. The proportion of women treated in teaching hospitals who underwent axillary surgery (65%) was twice that for women in non-teaching hospitals (32.5%, $P < 0.0001$). The proportion of women undergoing any

**Table I** Surgical treatment according to type of hospital

| | n | *Number of patients* | *Per cent of total* | *Mastectomy* | *Axillary surgery* |
|---|---|---|---|---|---|
| Teaching hospitals | 10 | 396 | (30%) | 167 (42%) | 258 (65%) |
| Non-teaching hospitals (>50 patients) | 5 | 376 | (28%) | 105 (28%) | 111 (30%) |
| Non-teaching hospitals (11–49 patients) | 15 | 454 | (34%) | 185 (41%) | 163 (36%) |
| Non-teaching hospitals (≤10 patients) | 19 | 98 | (7%) | 33 (34%) | 28 (29%) |
| Total | 49 | 1324 | | 490 (37%) | 560 (42%) |

axillary surgery decreased significantly over the study period from 114/231 (49%) in 1984 to 94/262 (36%) in 1988 ($P=0.003$). Axillary surgery rates remained constant over time within teaching hospitals, but decreased markedly in non-teaching hospitals (Table III).

The possibility was considered that variations in casemix between patients referred to teaching and non-teaching hospitals might account for the observed differences in surgical practice. This might apply particularly to residents of districts located an intermediate distance from a teaching hospital, for whom referral practice varied most widely. Further analyses were therefore undertaken, excluding the 352 residents of these districts. The mastectomy rate over the 5 year period in this subgroup was 42% for patients treated at teaching hospitals, compared with 32% for those treated at non-teaching hospitals ($P<0.004$). Similarly, 59% of teaching hospital patients underwent axillary surgery compared with 29% of non-teaching hospital patients ($P<0.0001$).

*Staging and tumour histology*

Extent of disease was recorded for 1556 (89%) patients, of whom 1161 (75%) were classified as having local disease, 298 (19%) local disease plus nodes and 97 (6%) metastatic disease at presentation. Extent of disease according to hospital type is shown in Table IV for the 1324 patients in group A. Patients treated in teaching hospitals were recorded as having 'local disease plus nodes' significantly more frequently than others ($P<0.0001$). Whether this reflects a true difference in casemix or simply reflects the greater use of axillary surgery in teaching hospitals and therefore more complete staging information is uncertain.

Tumour histology was recorded as non-specific in 608 of the 1757 cases (35%). Of these 'non-specific' diagnoses, 183 were made among the 272 patients whose tumours were not excised and were therefore presumably made on needle biopsy specimens. Among the 1324 patients in group A, 784 (59%) had ductal carcinoma, 195 (15%) had another specific diagnosis and 345 (26%) had a non-specific diagnosis. The rate of non-specific diagnosis was 17% in teaching hospitals, 20% at non-teaching hospitals treating more than 50 patients each and 38% in hospitals treating a smaller number of patients ($P<0.001$).

*Radiotherapy*

Radiotherapy was given to the breast and/or lymph nodes in 1116 (64%) of the 1757 patients. Within group A 170 (35%) of the 490 patients who underwent mastectomy also had radiotherapy. Of note was the fact that 149 (18%) of the 834 who underwent breast-conserving therapy were not recorded as having any radiotherapy.

*Systemic adjuvant therapy*

A total of 555 (42%) of the 1324 patients in group A received some form of adjuvant systemic therapy. Details of the adjuvant therapy administered are shown in Table V.

**Table II** Mastectomy rates: changes over time

| | *Teaching* | *Non-teaching* | *Total* |
|---|---|---|---|
| 1984 | 39/72 (54%) | 82/159 (52%) | 121/231 (52%) |
| 1985 | 44/103 (43%) | 76/180 (42%) | 120/283 (42%) |
| 1986 | 37/82 (44%) | 63/184 (34%) | 100/266 (38%) |
| 1987 | 24/75 (32%) | 53/207 (26%) | 77/282 (27%) |
| 1988 | 24/64 (38%) | 49/198 (25%) | 73/262 (28%) |
| Total | 168/396 (42%) | 323/928 (35%) | 491/1324 (37%) |

Decrease in mastectomy rates over time (test for trend): Teaching hospitals, $\chi^2=5.6$ on 1 d.f., $P=0.02$; non-teaching hospitals, $\chi^2=38.5$ on 1 d.f., $P<0.0001$.

**Table III** Axillary surgery: changes over time

| | *Teaching* | *Non-teaching* | *Total* |
|---|---|---|---|
| 1984 | 48/72 (67%) | 66/159 (42%) | 114/231 (49%) |
| 1985 | 66/103 (64%) | 65/180 (36%) | 131/283 (46%) |
| 1986 | 55/82 (67%) | 64/184 (35%) | 119/266 (45%) |
| 1987 | 48/75 (64%) | 54/207 (26%) | 102/282 (36%) |
| 1988 | 41/64 (64%) | 53/198 (27%) | 94/262 (36%) |
| Total | 258/396 (65%) | 302/928 (32.5%) | 560/1324 (42%) |

Change in axillary surgery rates over time (test for trend): Teaching hospitals, $\chi^2=0.07$ on 1 d.f., $P=0.79$; non-teaching hospitals, $\chi^2=12.8$ on 1 d.f., $P<0.0003$.

**Table IV** Extent of disease according to hospital type

| | *Teaching* | *Non-teaching* | *Total* |
|---|---|---|---|
| Local | 247 (69%) | 737 (82%) | 984 (78%) |
| Local and nodes | 94 (26%) | 134 (15%) | 228 (18%) |
| Metastatic | 17 (5%) | 29 (3%) | 46 (4%) |
| Unknown | 38 – | 28 – | 66 (–) |
| Total | 396 | 928 | 1324 |

$\chi^2=25.3$ on 2 d.f.; $P<0.0001$.

Table V Adjuvant therapy–according to hospital of first surgery

| | *Teaching* | *Non-teaching* | *Total* |
|---|---|---|---|
| Chemotherapy | 46 (12%) | 49 (5%) | 95 (7%) |
| Hormonal therapy | 89 (22%) | 336 (36%) | 425 (32%) |
| Combined therapy | 10 (3%) | 25 (3%) | 35 (3%) |
| Surgery only | 251 (63%) | 518 (56%) | 769 (58%) |
| Total | 396 | 928 | 1324 |

$\chi^2 = 34.6$ on 3 d.f.; $P < 0.0001$.

*Survival*

A total of 556 (32%) of the women were known to be dead at the end of follow-up (October 1991). The proportions of patients known to be dead according to year of registration were 42% for 1984, 36% for 1985, 38% for 1986, 26% for 1987 and 19% for 1988.

The influence of demographic factors (age and district of residence) and tumour-related factors (extent of disease and histology) on survival was assessed for the whole patient group ($n = 1702$), excluding those diagnosed through death certification only ($n = 55$). Both extent of disease ($P < 0.0001$) and histology ($P < 0.0001$) were highly significant predictors of survival, patients with localised disease and those with specific histological subtypes of breast cancer having the best outcome (Table VI). Neither age ($P = 0.13$) nor district of residence ($P = 0.14$) influenced survival significantly, though considerable differences in hazard ratios were observed between the districts with highest and lowest mortality (1.00 and 1.98 respectively).

A second analysis was undertaken restricted to the 1324 patients in group A. Demographic factors (age and district of residence) and type of hospital were included in the model. As differences in extent of disease and histological coding between teaching and non-teaching hospitals might be due to differences in surgical procedure and pathological expertise (see above), these factors were not included in this model. Among these surgically treated cases only age had a significant influence on survival, with patients under 35 years faring worse ($P = 0.02$).

### Discussion

Most reports on the management and outcome of patients with breast cancer in the UK relate to women treated in specialist centres or those treated in multicentre trials. One previous report compared the treatment and outcome of women managed at two centres where radiotherapy and chemotherapy were available, one in an urban teaching district, the other a rural non-teaching district (Basnett *et al.*, 1992). That study, involving a total of 999 women, 235 of whom were aged 50 years or less, appeared to show significantly worse survival for women treated in the non-teaching district (odds of death 1.46, $P = 0.0009$). The authors, however, advised that this finding should be treated with caution and recommended that cancer registries should be used as a tool for audit. A recent study from the Thames Cancer Registry examined the treatment given to 334 residents of the four Thames regions who were diagnosed in early 1990, 86 of whom were aged less than 50 years (Chouillet *et al.*, 1994). However, differences in management between treatment centres were not analysed and survival data were not shown.

In the current study, covering a defined geographical population, we have examined the treatment and outcome for women under 50 years. We were particularly interested in this age group as there is a strong rationale for undertaking axillary surgery in order to obtain prognostic information that may be of importance in giving advice related to the use of adjuvant systemic therapy. To the best of our knowledge this study is the first in which the number of patients treated

Table VI Survival analysis–Cox proportional hazards model

| *Variable* | *Hazard ratio* | *95% CI* | $\chi^2$ | *d.f.* | P-*value* |
|---|---|---|---|---|---|
| (a) *All patients (n = 1702)* | | | | | |
| District of residence | | | | | |
| Lowest hazard | 1.00 | | | | |
| Highest hazard | 1.98 | (1.19, 3.29) | 19.8 | 14 | 0.14 |
| Extent of disease | | | | | |
| Local | 1.00 | | | | |
| Local and nodes | 2.26 | (1.82, 2.80) | | | |
| Metastatic | 6.81 | (5.23, 8.88) | | | |
| Unknown | 1.42 | (1.02, 1.98) | 169.2 | 3 | 0.00001 |
| Histology | | | | | |
| Specific | 1.00 | | | | |
| Ductal | 1.62 | (1.17, 2.26) | | | |
| Non-specific | 2.13 | (1.52, 2.99) | 22.7 | 2 | 0.00001 |
| Age | | | | | |
| Under 35 | 1.00 | | | | |
| 35 and over | 0.80 | (0.60, 1.06) | 2.3 | 1 | 0.13 |
| (b) *Surgical cases only (n = 1324)* | | | | | |
| District of residence | | | | | |
| Lowest hazard | 1.00 | | | | |
| Highest hazard | 2.66 | (1.37, 5.17) | 17.5 | 14 | 0.23 |
| Hospital type | | | | | |
| Non-teaching | 1.00 | | | | |
| Teaching | 1.27 | (0.93, 1.74) | 2.2 | 1 | 0.14 |
| Age | | | | | |
| Under 35 | 1.00 | | | | |
| 35 and over | 0.67 | (0.49, 0.93) | 5.3 | 1 | 0.02 |

in different centres, and the type of hospital (teaching, non-teaching or private) has been assessed in relation to survival. This may be of particular relevance if the recent recommendations of the Expert Advisory Group on Cancer to the Chief Medical Officers of England and Wales are to be implemented (Expert Advisory Group on Cancer, 1995). That report recommends the establishment of a network of 'cancer centres' and 'cancer units' throughout the UK, and that a hospital should seek to function as a cancer unit only if the volume of work related to each cancer site is sufficient to maintain subspecialisation.

Although it is impossible to be certain of the completeness of ascertainment of cases by TCR, estimates from the Registry place completeness for breast cancer at all ages at over 86% of all cases diagnosed (J Bullard, personal communication). There is no reason to believe that there are any inherent biases in the completeness of reporting from different hospitals. The completeness of the treatment data is also good for most items. The quality of registry data is, however, dependent on the quality of hospital case records. The study by Chouillet *et al.* (1994) showed that retrospective reconstruction of staging (I–IV) is only possible in about 50% of cases even after detailed scrutiny of case records. We therefore used the staging classification adopted by Thames Cancer Registry, which is based on pathological information when available and clinical data for the remainder. This undoubtedly underestimates the true incidence of node positivity, which might be expected to be approximately 50%, compared with 19% classified as having 'local disease and nodes' in the current study. We also adopted a simple classification of treatments to minimise errors due to differences in recording of surgical procedures by surgeons.

Women in the UK with suspected breast cancer are normally referred to a general surgeon for assessment. As shown in this study, breast surgery was undertaken in 42 NHS hospitals in SETRHA during the 5 year study period. The short study period of only a few weeks covered in the report by Chouillet *et al.* (1994) may have led to a considerable underestimate of the total number of hospitals managing primary breast cancer (reported as 81 for the four Thames regions) as those hospitals managing only a few cases annually may well not have diagnosed any patients during that period.

In the current study almost 90% of the patients who underwent surgery received this in NHS hospitals within the region or in teaching hospitals in adjacent regions. Of these, 58% received their surgical treatment in a teaching hospital or in one of five non-teaching hospitals with a relatively large breast cancer practice (i.e. on average more than ten cases in this age group per annum). A further 34% of patients underwent surgery in a total of 15 hospitals, each of which treated between two and ten patients in this age group annually. The remaining 7% of the patient population underwent surgery in a total of 19 hospitals, each of which dealt with an average of less than two such patients annually. Although these figures apply only to residents of SE Thames region, it is unlikely that significant cross boundary flows occurred with patients being referred to SE Thames hospitals other than to teaching hospitals.

Referral to a teaching hospital or to a non-teaching hospital depended largely on geographical access. Almost all patients living in districts situated more than 20 miles from a teaching hospital underwent primary surgery in a non-teaching hospital. Conversely, the large majority of women living in the districts served directly by a teaching hospital underwent treatment there. For women resident in the four districts situated an intermediate distance from a teaching hospital it is possible that referral may have been affected by casemix (e.g. tumour size). However, it seems more likely that individual GPs in these districts selectively refer the majority of women with breast problems to one preferred centre. In any case, when women in these four districts were excluded from the analysis, the differences in surgical practice observed between hospital types remained highly significant.

Does this wide dispersal of patients between hospitals affect either the care that they receive or their long-term survival? The King's Fund consensus statement (1986) concluded that although there is no evidence that survival is any better in specialist units than in general hospitals, a strong case can be made for grouping together the services for women with breast cancer. This was based on the assumption that surgeons with no special interest in breast cancer are less likely to be aware of trial results and other advances, and may be less skilled in appreciating the woman's need for information and psychological support. It must have been difficult, if not impossible, for the 34 hospitals identified in this study as managing only a small number of patients each year to provide care in the setting of a multidisciplinary breast clinic. The King's Fund consensus statement argues that such a clinic should involve a surgeon, pathologist, trained nurse counsellor, and a radiotherapist and/or medical oncologist. In each district a psychiatrist should be attached to the breast team. This multidisciplinary approach would help to ensure that appropriate treatments are considered for every patient. It would also serve to meet the women's information, psychological and practical needs on a more comprehensive basis. The advantages of multi-disciplinary care within specialist breast clinics have recently been stressed in a report from the British Breast Group (1994).

In keeping with the assertion made in the consensus statement, we observed no difference in survival according to the number of patients treated in a particular hospital. However, meaningful survival comparisons require knowledge of the casemix in different hospitals that is not available in the TCR data. The teaching hospitals appeared to have a lower proportion (69%) of localised cases than district general hospitals (79%), but this may reflect either the use of more axillary nodal surgery in teaching hospitals, or more advanced cases being referred there. The Registry is now collecting data on tumour size and the number of nodes sampled in order to provide more accurate information on casemix. The influence of tumour size on outcome has been clearly demonstrated in a study involving almost 25 000 women with breast cancer (Carter *et al.*, 1989).

As far as the assessment of women with breast cancer is concerned, involvement of axillary lymph nodes is well recognised as the most important determinant of prognosis, followed by histological type and grade (Carter *et al.*, 1989; Bloom and Richardson, 1957; O'Reilly *et al.*, 1990). Information regarding axillary nodal status may be of particular importance in women under 50 years, as this may be used in deciding whether to recommend adjuvant chemotherapy. The consensus statement clearly recommended that axillary nodes should be sampled at the time of breast surgery. In practice, only 42% of women with operable breast cancer in this study were recorded as having any form of axillary surgery. Axillary surgery was performed less frequently in district general hospitals and the proportion of patients receiving such assessment actually fell after the publication of the consensus statement. The less frequent use of axillary surgery in non-teaching hospitals is in direct contrast to the findings reported by Basnett *et al.* for the same age group (1992). The low overall rate of axillary surgery is, however, similar to that reported by other studies (Chouillet *et al.*, 1994; Basnett *et al.*, 1992). It should be noted that some underrecording of axillary surgery by TCR may have affected this study, as this has been observed when Registry data are compared with case records for audit purposes (AM Chouillet, personal communication). We have no reason, however, to believe that this would affect the trends noted over time or differences observed between hospital types. The low rates of axillary surgery in this and other studies do, however, call into question the reliability of information gained from questionnaire surveys as a guide to what occurs in clinical practice (Morris *et al.*, 1989, 1992). For example, in reply to a questionnaire in 1992, 77% of surgeons recommended either axillary dissection or sampling (Morris *et al.*, 1992).

In addition to the inadequate assessment of nodal status, this study has also shown that the prognostic information related to histological type is also less frequently available among patients treated in hospitals with a smaller breast cancer practice. Information related to tumour grade was not available for this study. However, the larger proportion of cases with non-specific morphology codes in hospitals treating smaller numbers of patients suggests that this prognostic information is also frequently not available.

It has now been demonstrated in several randomised controlled trials that, for appropriately selected cases, long-term survival following breast conserving therapy is equivalent to that observed following mastectomy. Preliminary reports from two of these studies (Veronesi *et al.*, 1981; Sarrazin *et al.*, 1983) were published before the start of our study period and a major report from the USA was published in 1985 (Fisher *et al.*, 1985). The proportion of women under 50 years in SETRHA receiving breast conservation therapy of any form increased during the study period from 48% to 72% of operable cases, with higher rates consistently being observed in non-teaching hospitals. Without knowledge of tumour size or multifocality, it is difficult to judge whether such treatment was appropriate. Nor is it possible to assess what choice was offered to individual patients. However, it is clear that surgeons working outside teaching hospitals have adopted the use of breast conservation therapy more rapidly and for a larger proportion of their patients than have their teaching hospital colleagues.

Knowledge of the reduction in relapse and death rates achievable in women under 50 years with systemic adjuvant therapy increased markedly during the 1980s, with several reports being published by 1985 (Rossi *et al.*, 1981; Fisher *et al.*, 1983; Anonymous, 1984; Baum *et al.*, 1983, 1985). The King's Fund consensus statement made it clear that (i) combination chemotherapy reduces the death rate in node-positive patients aged less than 50 years; (ii) destroying ovarian function had a similar effect on mortality; (iii) there was some evidence of a reduced relapse rate with tamoxifen.

In practice, the use of adjuvant chemotherapy was very limited in SE Thames at least up to 1988. Indeed, most treated cases were confined to one hospital in which trials of chemotherapy were being conducted. Tamoxifen was, however, quite widely used, particularly in district general hospitals. It should be noted that several hospitals in SE Thames participated in the NATO and CRC adjuvant therapy studies between 1977 and 1985 and physicians were thus familiar with the use of tamoxifen (Baum *et al.*, 1983; CRC Adjuvant Breast Trial Working Party, 1988). Patients in SE Thames may thus have benefited from the administration of tamoxifen, even though the objective evidence supporting its effect on survival in premenopausal women has only become clear-cut more recently (Scottish Cancer Trials Office, 1987; Early Breast Cancer Trialists' Collaborative Group, 1992). The findings of the current study can be compared with those for 383 patients of all ages treated at the Middlesex and University College Hospitals in 1986 (McCarthy and Bore, 1991). As in our study, the authors noted that a significant number of patients received lumpectomy without radiotherapy. Chemotherapy was given to 27% and tamoxifen to 26% of women under 50 years (compared with 12% and 22% respectively in our teaching hospital group).

In conclusion, this study has demonstrated that primary treatment for women aged less than 50 years is provided in a multitude of different hospitals, the majority of which treat only very limited numbers of patients. Treatment given to patients varied significantly between hospitals. While this has not been shown to have an adverse effect on survival, a high proportion of women managed outside teaching hospitals are receiving a form of surgical management that has never been validated by randomised controlled trials. The lack of pathological information related to nodal status almost certainly leads to inappropriate recommendations regarding adjuvant treatment.

**Acknowledgements**
We are grateful to the Thames Cancer Registry for providing the data for this study. MA Richards was funded by the Imperial Cancer Research Fund. K Tilling and J Barton are funded by the Department of Health.

### References

ANONYMOUS. (1984). Review of mortality results in randomised trials in early breast cancer. *Lancet*, **2**, 1205.

BASNETT I, GILL M AND TOBIAS JS. (1992). Variations in breast cancer management between a teaching and a non-teaching district. *Eur. J. Cancer*, **28A**, 1945–1950.

BAUM M, BRINKLEY DM, DOSSETT JA, MCPHERSON K, PATTERSON JS, RUBENS RD, SMIDDY FG, STOLL BA, WILSON A, LEA JC, RICHARDS D AND ELLIS SH. (1983). Controlled trial of tamoxifen as adjuvant agent in management of early breast cancer. *Lancet*, **1**, 257–261.

BAUM M, BRINKLEY DM, DOSSETT JA, MCPHERSON K, PATTERSON JS, RUBENS RD, SMIDDY FG, STOLL BA, WILSON A, RICHARDS D AND ELLIS SH. (1985). Controlled trial of tamoxifen as single adjuvant agent in management of early breast cancer. *Lancet*, **1**, 836–840.

BLOOM HJG AND RICHARDSON WW. (1957). Histological grading and prognosis in breast cancer. *Br. J. Cancer*, **5**, 173–183.

BRITISH BREAST GROUP. (1994). Provision of breast services in the UK: the advantages of specialist breast units. (*The Breast*).

CARTER CL, ALLEN C AND HENSON DE. (1989). Relationship of tumour size, lymph node status and survival in 24,740 breast cancer cases. *Cancer*, **63**, 1170–1178.

CHOUILLET AM, BELL CMJ AND HISCOX JG. (1994). Management of breast cancer in Southeast England. *Br. Med. J.*, **308**, 168–171.

CRC ADJUVANT BREAST TRIAL WORKING PARTY. (1988). Cyclophosphamide and tamoxifen as adjuvant therapies in the management of breast cancer. *Br. J. Cancer*, **57**, 604–607.

DIXON WJ. (1990). *BMDP Statistical Software Manual*, Vol 2. pp. 739–806. University of California Press: Berkeley.

EARLY BREAST CANCER TRIALISTS' COLLABORATIVE GROUP. (1992). Systemic treatment of early breast cancer by hormonal, cytotoxic or immune therapy. *Lancet*, **339**, 1–15, 71–85.

EXPERT ADVISORY GROUP ON CANCER. (1995). *A Policy Framework for Commissioning Cancer Services*. Department of Health.

FISHER B, BAUER M, MARGOLESE R, POISSON R, PILCH Y, REDMOND C, FISHER E, WOLMARK N, DEUTSCH M, MONTAGUE E, SAFFER E, WICKERHAM L, LERNER H, GLASS A, SHIBATA H, DECKERS P, KETCHAM A, OISHI R AND RUSSELL I. (1985). Five year results of a randomised clinical trial comparing total mastectomy and segmental mastectomy with or without radiation in the treatment of breast cancer. *N. Engl. J. Med.*, **312**, 665–673.

FISHER ER, REDMOND C AND FISHER B. (1983). Pathologic findings from the National Surgical Adjuvant Breast Project. Relationship of chemotherapeutic responsiveness to tumour differentiation. *Cancer*, **51**, 181–191.

KING'S FUND FORUM (1986). Consensus devlopment conference: treatment of primary breast cancer. *Br. Med. J.*, **293**, 946–947.

LICHTER AS, LIPPMAN ME, DANFORTH JR DN, D'ANGELO T, STEINBERT SM, DEMOSS E, MACDONALD HD, REICHERT CM, MERINO M, SWAIN SM, COWAN K, GERBER LH, BADER JL, FINDLAY PA, SCHAIN W, GORRELL CR, STRAUS K, ROSENBERG SA AND GLATSTEIN E. (1992). Mastectomy versus breast conserving therapy in the treatment of stage I and II carcinoma of the breast: A randomised trial at the National Cancer Institute. *J. Clin. Oncol.*, **10**, 976–983.

MCCARTHY M AND BORE J. (1991). Treatment of breast cancer in two teaching hospitals: a comparison with consensus guidelines. *Eur. J. Cancer*, **27**, 579–582.

MORRIS J, ROYLE GT AND TAYLOR I. (1989). Changes in the surgical management of early breast cancer in England. *J. R. Soc. Med.*, **82**, 12–13.

MORRIS J, FARMER A AND ROYLE G. (1992). Recent changes in the surgical management of T1/2 breast cancer in England. *Eur. J. Cancer*, **28A**(10), 1709-1712.

OFFICE OF POPULATION CENSUSES AND SURVEYS. (1994). Cancer statistics registrations: cases of diagnosed cancer registered in England and Wales 1988 (series MB1 no 21). HMSO: London.

OFFICE OF POPULATION CENSUSES AND SURVEYS. (1993). Mortality Statistics, Cause. England and Wales (series DH2 no 18). HMSO: London.

O'REILLY SM, CAMPLEJOHN RS, BARNES DM, MILLIS RR, ALLEN D, RUBENS RD AND RICHARDS MA. (1990). DNA index, S phase fraction, histological grade and prognosis in breast cancer. *Br. J. Cancer*, **61**, 671-674.

ROSSI A, BONADONNA G, VALAGUSSA P AND VERONESI U. (1981). Multimodal treatment in operable breast cancer: Five year results of the CMF programme. *Br. Med. J.*, **282**, 1427-1431.

SARRAZIN D, LE M, FONTAINE MF AND ARRIAGADA R. (1983). Conservative treatment versus mastectomy in T1 or small T2 breast cancer. A randomised clinical trial. In *Conservative Management of Breast Cancer*. Harris JR, Hellman S and Silen W. (eds). pp. 101-111. JB Lippincott: Philadelphia.

SCOTTISH CANCER TRIALS OFFICE. (1987). Adjuvant tamoxifen in the management of operable breast cancer: The Scottish trial. *Lancet*, **2**, 171-175.

THAMES CANCER REGISTRY. (1993). *Cancer in South East England*. Thames Cancer Registry: Sutton.

VAN DONGEN JA, BARTELINK H, FENTIMAN IS, LERUTE T, MIOLET S, OLDHUIS G, VAN DER SCHUEREN E, SILVESTER R, TONG D, WINTER J AND VAN ZIJL K. (1991). Randomised clinical trial to assess the value of breast conserving therapy (BCT) in stage I and stage II breast cancer: EORTC 10801. Proceedings of EORTC 5th Breast Cancer Working Conference, Leuven, September 1991.

VERONESI U, SACCOZZI R, DEL VECCHIO M, BANFI A, CLEMENTE C, DE LENA M, GALLUS G, GRECO M, LUINI A, MARUBINI E, MUSCOLINO G, RILKE F, SALVADORI B, ZECCHINI A AND ZUCALI R. (1981). Comparing radical mastectomy with quadrantectomy, axillary dissection, and radiotherapy in patients with small cancers of the breast. *N. Engl. J. Med.*, **305**, 6-11.